# Development of Allogeneic Stem Cell-Based Platform for Delivery and Potentiation of Oncolytic Virotherapy

**DOI:** 10.3390/cancers14246136

**Published:** 2022-12-13

**Authors:** Duong Hoang Nguyen, Thomas Herrmann, Barbara Härtl, Dobrin Draganov, Ivelina Minev, Forrest Neuharth, Alberto Gomez, Ashley Alamillo, Laura Edith Schneider, Daniela Kleinholz, Boris Minev, Antonio F. Santidrian

**Affiliations:** 1 Calidi Biotherapeutics, San Diego, CA 92037, USA; 2 StemVac, D-82347 Bernried, Germany; 3 Department of Radiation Medicine and Applied Sciences, University of California, San Diego, CA 92093, USA

**Keywords:** SNV1, Supernova, CAL1, vaccinia virus, oncolytic virus, cell-based platform, cancer therapy

## Abstract

**Simple Summary:**

The therapeutic potential of the oncolytic virotherapy is severely restricted by multiple innate and adaptive immune barriers. Here, we describe how the Supernova (SNV) cell-based oncolytic platform can be utilized to generate off-the-shelf products for cancer treatments. CAL1 vaccinia virus was loaded into adipose-derived mesenchymal stem cells to generate SNV1. SNV1 shows more resistant to rapid inactivation by humoral immune system as compared to naked CAL1 virus leading to a significant and robust improvement of oncolytic virus therapeutic efficacy in multiple animal models. Particularly, SNV1 provided instantly active viral particles for immediate infection and simultaneous release of therapeutic proteins in the injected tumors, potentially improving virus-based cancer therapies in the clinic.

**Abstract:**

We describe the repurposing and optimization of the TK-positive (thymidine kinase) vaccinia virus strain ACAM1000/ACAM2000™ as an oncolytic virus. This virus strain has been widely used as a smallpox vaccine and was also used safely in our recent clinical trial in patients with advanced solid tumors and Acute Myeloid Leukemia (AML). The vaccinia virus was amplified in CV1 cells and named CAL1. CAL1 induced remarkable oncolysis in various human and mouse cancer cells and preferentially amplified in cancer cells, supporting the use of this strain as an oncolytic virus. However, the therapeutic potential of CAL1, as demonstrated with other oncolytic viruses, is severely restricted by the patients’ immune system. Thus, to develop a clinically relevant oncolytic virotherapy agent, we generated a new off-the-shelf therapeutic called Supernova1 (SNV1) by loading CAL1 virus into allogeneic adipose-derived mesenchymal stem cells (AD-MSC). Culturing the CAL1-infected stem cells allows the expression of virally encoded proteins and viral amplification prior to cryopreservation. We found that the CAL1 virus loaded into AD-MSC was resistant to humoral inactivation. Importantly, the virus-loaded stem cells (SNV1) released larger number of infectious viral particles and virally encoded proteins, leading to augmented therapeutic efficacy in vitro and in animal tumor models.

## 1. Introduction

Multiple clinical trials using a variety of replication-competent vaccinia virus strains (VACV) showed safety and signals of therapeutic potential in patients with various types of cancer [1,2,3,4]. To reduce the risk for cancer patients, some highly virulent VACV strains, like Western Reserve, were genetically engineered to attenuate the virus and improve tumor selectivity or safety in clinical trials. This method, however, could potentially compromise virus potency [5] and therapeutic efficacy [6,7,8].

The availability of known naturally tumor selective VACV strains used as smallpox vaccines with already established safety profiles provides an opportunity to develop oncolytic VACV platforms without the need for additional attenuating modifications or improved tumor selectivity. The initial evidence demonstrating the therapeutic efficacy and safety of non-genetically attenuated wild-type VACV in the treatment of cancer came from the off-label use of the virus in metastatic melanoma patients. As early as 1960, reports suggested that the polyclonal smallpox vaccine Dryvax^®^ was effective against metastatic melanoma, and that this vaccine could safely be injected directly into cutaneous lesions [9]. Several subsequent studies, using various globally approved smallpox vaccines, demonstrated the feasibility and safety of using non-genetically attenuated VACV to treat cancer patients even in the absence of protective prophylactic vaccination [10,11].

We have recently shown the safety and feasibility of repurposing the FDA-approved smallpox vaccine ACAM2000, a TK-positive vaccinia virus, to treat patients with advanced solid tumors and AML [1]. ACAM2000 was originally derived from ACAM1000, which is a reduced-virulence clone derived from the parental vaccine Dryvax^®^ [12,13]. ACAM1000 and ACAM2000 have the same DNA sequence but were manufactured in MRC-5 or Vero cells, respectively [14], reflecting the different cell culture propagation. These two viral preparations have shown equivalent safety profiles [15]. ACAM1000/ACAM2000™ has a publicly available sequenced genome that contains 200 open reading frames (ORFs) [13,14]. Key naturally occurring alterations in their genome explain the reduced virulence of ACAM1000/ACAM2000. In particular, a truncation of ankyrin-repeat ortholog of VACV– and cowpox–like protein, a short form of the thymidylate kinase gene may contribute to its naturally reduced virulence. The truncations to the tumor necrosis factor receptor and the interferon α/β binding protein, which are important immunomodulators, might further contribute to the reduced virulence of this VACV strain [14]. Type I interferons (IFN) have been reported to inhibit viral replication in normal cells, while permitting viral replication in cancer cells due to their intrinsic defects in IFN signaling [16]. Thus, the naturally occurring truncations in the interferon α/β binding protein can improve the tumor selectivity of the virus by attenuating virus infection and spread in normal cells and tissues with functional IFN responses, but not in cancer cells with dysfunctional antiviral mechanisms [7,17].

Although ACAM1000/ACAM2000™ vaccinia virus could be repurposed for anti-cancer therapy, a major remaining challenge is maximizing the anti-tumor efficacy by establishing effective delivery platforms. In the clinic, oncolytic virus treatment encounters critical initial barriers including complement, anti-viral antibodies, and innate immune cell inactivation, which dramatically decreases its therapeutic potential. As many other oncolytic viruses, oncolytic vaccinia virus shares several critical limitations related to viral delivery, antiviral immunity, resistance, and spreading [18]. Thus, the clinical efficacy of oncolytic viruses depends on the successful delivery of the virotherapy agent to the targeted tumor and the efficient initial viral amplification at the tumor sites [19]. To repurpose ACAM1000/ACAM2000™ as a potent oncolytic virus, vaccinia virus should be protected from the patients’ immune system and effectively delivered to the tumor sites.

To overcome these limitations researchers have been using cell-based vehicles to protect and deliver oncolytic viruses systemically [20,21,22]. In our recent clinical trial, we showed that adipose-derived autologous stem cells (Stromal vascular fraction (SVF)) have the potential to protect and deliver oncolytic vaccinia virus [1]. The trial confirmed the safety of the oncolytic virus ACAM2000 even in patients with advanced solid tumors and AML. We also observed anti-tumor effects in several patients, including complete responses especially in combination with checkpoint inhibitors. However, this clinical trial also revealed significant shortcomings of using autologous stromal vascular cells to protect the oncolytic virus, as the autologous SVF cells are not a uniform cell population, containing only a small portion of “virus-loadable” cells (1–8%) of the total SVF which is patient-dependent [23]. The use of allogeneic adipose-derived stem cells as proposed in this study will contain >98% virus-loaded cells. Additional advantages, of using stem cells in allogeneic settings are (i) elimination of the need to subject each patient to a liposuction procedure, which has a potential to cause infection and other complications, especially in immunocompromised patients with advanced tumors (ii) higher manufacturing scalability under cGMP, and (iii) an allogeneic stem-cell based product would have longer shelf life allowing to the same product to be injected multiple times to the same patient and to treat many patients with multiple doses. Our in vitro studies further suggested that stem cells-based systemic delivery of oncolytic vaccinia virus in allogeneic setting is feasible but might require the use of matched allogeneic stem cells to overcome existing cellular immune barriers which might be critical therapeutic success [24]. In this study we describe the development of a new allogeneic stem cell-based platform design for intratumoral (i.t) administration, called Supernova (SNV), where the virus was protected from humoral immunity, amplified, and potentiated inside the stem cells minimizing its clearance by the immune system. Direct i.t. administration of immune oncology agents often offers a better and more successful translation to the clinic since it efficiently targets the tumor and avoids the significant dilution associated with intravenous approaches [25]

We found that SNV drastically enhanced the therapeutic efficacy of CAL1 following i.t administration. We also report that this virus can also be used as a backbone to generate engineered recombinant viruses armed with therapeutic genes, which can be delivered using SNV platform to be therapeutically effective in patients.

## 2. Materials and Methods

### 2.1. Next Generation Sequencing (NGS)

NGS sequencing was performed LGC Genomics GmbH, (Berlin, Germany) using 300 bp paired end read Illumina MiSeq V3 platform. DNA purification was performed starting from 100 µL of purified Vaccinia Virus CAL1 using QIAamp Mini Kit #69506 (Qiagen, CA, USA) according to the manufacturer’s guidelines. DNA was then quantified by NanoDrop™ Lite Spectrophotometer (Thermo Fisher Scientific, Waltham, MA, USA) and 200 nanogram was used to generate MiSeq-DNA shotgun library (Illumina Inc., San Diego, CA, USA). Deep sequencing was performed on the MiSeq V3 platform. (Illumina Inc., San Diego, CA, USA). Demultiplexing of all library groups, clipping and trimming were performed by Illumina bcl2fastq 2.17.1.14 software prior to data analysis which was performed using Geneious prime software v. 2020 (Biomatters, Inc., San Diego, CA, USA).

### 2.2. Cell Culture

PC3, MDA-MB-231, RPMI-7951, FaDu, TRAMP-C2, RM-1, B16F-10, EMT-6, CT-26, DU-145, E006AA, HPrEC, and CV-1 cell lines were purchased from ATCC (Manassas, VA, USA). PC3, MDA-MB-231, EMT6 and CV1 cells were maintained in Dulbecco’s Modified Eagle Medium (DMEM, Gibco, Carlsbad, CA, USA) with 4.5 g/L glucose, 100 U/mL Pen/Strep (Gibco), 2 mM L-glutamine (Gibco), and 10% fetal bovine serum (FBS, Omega Scientific, Tarzana, CA, USA). DU145 and RPMI-7951 cells were maintained in low-glucose Dulbecco’s modified Eagle’s medium (DMEM, Gibco), supplemented with 2 mM L-glutamine (Gibco), 100 U/mL Pen/Strep (Gibco), and 10% FBS (Omega Scientific). E006AA cells were maintained in DMEM (Gibco) supplemented with 10% FBS (Omega Scientific). HPrEC cells were maintained in Prostate Epithelial Cell Basal Medium supplemented with 6 mM L-glutamine, 0.4% extract P, 1.0 µM epinephrine, 0.5 ng/mL rh TGF-α, 100 ng/mL hydrocortisone, 5 µg/mL rh insulin, and 5 µg/mL apo-transferrin through the addition of a Prostate Epithelial Cell Growth Kit. B16-F10, CT26, and RM-1 cells were maintained in high-glucose DMEM with 4.5 g/L glucose, 1.1 g/L sodium pyruvate (Gibco), 2 mM L-glutamine (Gibco), 10% FBS (Omega Scientific), 100 U/mL Pen/Strep (Gibco). FaDu cells were maintained in Eagle’s Minimum Essential Medium (EMEM) (ATCC), 10% FBS (Omega Scientific), 100 U/mL Pen/Strep (Gibco). TRAMP-C2 cells were maintained in DMEM with 4.5 g/L glucose supplemented with 5% Nu-Serum IV (Corning), 5% FBS (Corning), 2 mM L-Glutamine (Gibco), 100 U/mL Pen/Strep (Gibco), 0.005 mg/mL Bovine Insulin (Sigma, Fukushima, Japan) and 10 nM Dehydroisoandrosterone (Sigma). Cells were grown in an incubator at 37 °C, 5% CO_2_, and in a humidified atmosphere.

### 2.3. Virus Amplification Assay

CAL1 went through several rounds of amplification on CV-1 cells. CAL2, which has TurboFP635 inserted at an intergenic site, was genetically engineered from CAL1 as described below. Cells were plated in 24-well dishes at 90–100% confluency in 0.5 mL of growing media. At 4–5 h after plating, cells were synchronously infected in duplets with CAL1 at MOIs 0.01 and 0.1. At 24 h post-infection, two wells of each cell line and MOI with infected cell samples were harvested by scraping with the rubber head of a 1 mL syringe, transferred to a 1.5 mL tube, and stored at −20 °C until analysis via plaque assay. The same steps were repeated for dishes at 48, 72 and 96 h. Prior to performing plaque assays, samples were freeze–thawed three times.

### 2.4. Adipose-Derived Stem Cell Generation and Characterization

Adipose-derived mesenchymal stem cells (AD-MSCs) (also known as medicinal stem cells or mesenchymal stroma cells [26]) were generated from mini liposuctions by a specialized GMP stem cell manufacturing company called Vetstem Biopharma (Poway, CA, USA) under IRB approval#: IRCM-2018-196. Briefly, fresh stromal vascular fraction (SVF) isolated from fat tissue from 10 healthy donors were collected and plated overnight using hSTEM media supplemented with growth factor cocktail (Vetstem Biopharma, Poway, CA). Next day, media supernatant was washed to remove unattached cells and debris. Media was changed every 3–4 days until the mesenchymal stem cells started to grow and reached 80% confluency. AD-MSCs were then expanded in attachment and verified the expression of surface markers by flow cytometry (Attune NxT Flow Cytometer, Thermo Fisher Scientific, Waltham, MA, USA), using BD Stemflow™ hMSC Analysis kit (BD Biosciences, San Jose, CA, USA, Cat# 562245) and manual, and analyzed by Flowjo v.10 software (Ashland, OR, USA) as described before [23,27,28]. AD-MSCs generated in this study were positive (>85%) for CD73, CD90, and CD105, but negative (<5%) for CD34, CD45, CD11b, CD19, and HLA-DR. One cell line from one donor, VP01, was selected to generate SNV1 due to the high proliferation profile, >0.7 population doubling per day (PD/d). After incubation of AD-MSCs with CAL1 virus, the SNV1 cells were frozen down at concentration of 10 cells/mL per vial using commercial freezing media CryoStor^®^ CS5 (Durham, NC, USA, Cat# 205102) in Corning™ CoolCell™ LX Cell Freezing Vial Containers (Tewksbury, MA, USA, Cat. 07-210-003). SNV1 cells were stored in Liquid Nitrogen.

### 2.5. SNV1 Production

MSCs were thawed and cultured for 2 passages in stem cell media. Once the cells were about 90% confluent, cells were trypsinized using TrypLE™ Express (Life Technologies, Carlsbad, CA, USA; Cat# 12604021) for 10 min in a 37 °C incubator. Cells were counted and mixed with CAL1 virus using multiplicity of infection (MOI) < 1. The infected cells were then incubated for 20–24 h in CO_2_ incubator at 37 °C. At the time of collection, the cells were washed, trypsinized, counted with NucleoCounter^®^ NC-200™ (ChemoMetec, La Jolla, CA, USA), subsequently cryopreserved at concentration of 10 × 10^6^ cells per mL of CryoStor^®^ CS5, and named SNV1. A frozen vial of SNV1 was thawed, counted with NucleoCounter^®^ NC-200™ (ChemoMetec). Additionally, viability of the cells was analyzed using NucleoCounter^®^ NC-200™ (ChemoMetec) and amount of viral particle per cell (PFU/cell) using plaque assays.

### 2.6. Plaque Assay

CV-1 cells were plated in 24-well plates at 2 × 10^5^ cells/well one day before the assay. Samples were serially diluted 1:10 in Dulbecco’s modified Eagle’s medium supplemented with 2 % Fetal Calf Serum (FCS) (DMEM2) and 200 μL of each dilution or DMEM2 only was aliquoted in duplets into wells. Following an incubation of 1–2 h, 0.5 mL of carboxymethyl cellulose overlay media was added to each well and the plates were incubated for 48 +/− 6 h at 37 °C and 5% CO_2_. Crystal violet was used to stain cells for 1–4 h. Once staining was complete, the stain solution was aspirated; wells were washed twice for 1 min with 1 mL water and air-dried. Plaques were quantified macroscopically or by microscopic evaluation, if needed.

### 2.7. Serum Collection

Blood samples were collected from healthy donors under approved IRB #IRCM-2019-203 (Protocol #CB-IR-002) using BD Vacutainer Plus plastic serum tube (BD Biosciences, San Jose, CA, USA, Cat# 367820) (10 mL/tube, 2 tubes for each donor). Blood samples were incubated for 30–45 min at room temperature (RT) in an upright position, then were centrifuged at 2000× *g* for 15 min at RT. Supernatant (serum) was collected, aliquoted, and stored at −80 °C. Each sample was assigned an identifying number (ex.: CBD01, CBD02…). The Human AB Serum was obtained from Valley Biomedical (Winchester, VA, USA, Cat# H1022).

### 2.8. Vaccinia Virus Neutralizing Antibody Analysis

The CV-1 cells (2 × 10^4^) were seeded on 96-well electrode microplate in DMEM2 media and placed in the xCELLigence RTCA for 24 h. Human serum donors were heat inactivated at 65 °C for 30 min. Serial dilutions of human sera were mixed with 20 PFU CAL1 virus (1:1) and incubated for 1 h at 37 °C with agitation every 15 min. Once the incubation in water bath process was completed, the mixture of CAL1 virus and serum was applied to CV1 cell monolayers measuring the cell index every 15 min using xCELLigence RTCA MP. Wells with or without CAL1 virus served as positive or negative controls. Percentage of cell cytolysis was calculated according to following formular:

% cytolysis = (CI control − CI CAL1 virus)/CI control × 100 (CI = cell index)

Percentage of 80% or 100% blockage of viral infectivity was calculated by subtraction % cytolysis from 100. Results reflect the average of 3 replicate wells.

### 2.9. Animal Tumor Models

Mice were handled in accordance to approved protocols by the Institutional Animal Care and Use Committee implemented by Explora Biolabs (San Diego Science Center, San Diego, CA). Athymic nude male mice (Charles River Laboratories, Wilmington, MA, USA) from 4-6 weeks were inoculated with 2.5 × 10^6^ of PC3 cells subcutaneously in 100 µL PBS on the right flank. When the tumors reached an overall geomean volume of 200 mm^3^, mice were randomized based on tumor size and stratified into treatment groups of 1 × 10^6^ SNV1 (equivalent to or 1 × 10^7^ PFU), 1 × 10^7^ PFU CAL1, or PBS only (n = 10/group, 50 µL per injection). Treatment was delivered intratumorally, and tumor size was measured twice a week. Female BALB/c mice were inoculated with 2.5 × 10^5^ murine colon cancer cells (CT26) subcutaneously in the right flank. When tumor sizes were around 50 mm^3^, mice were intratumorally treated with 1 × 10^7^ PFU of CAL1 vaccinia virus or 1 × 10^6^ SNV1 cells for three times, every 2 days (n = 6–7/group). Tumor volume was calculated following this formula:

Tumor Volume = height × width × length × 0.5

### 2.10. Real Time Cell Analysis of Virus Mediated Cytolysis

Human cancer cell lines: PC3, RPMI-7951, MDA-MB-231, FaDu, or mouse cancer cell lines: TRAMPC2, RM1, B16F10, EMT6 or CT26 were cultured a day before to form a monolayer on 96-well electronic microplates. Cells were infected with CAL1 or SNV1 at different MOIs in the presence of 20% human sera or fetal bovine serum in a final volume of 200 µL/well. Cell attachment and proliferation from selected wells of the plate were monitored and recorded every 15 min using the RTCA MP. Cytolysis was analyzed in real-time or at 24-, 48-, 72-, and 96-h post-infection using RTCA software Pro 2.3.2 (Agilent, CA, USA). A parameter termed cell index was used to quantify cell attachment status based on detected cell-electrode impedance. Results reflect the average of 3 replicate wells.

[% of cytolysis = (Cytolysis Index control − Cytolysis Index CAL1 virus)/CytoIysis Index control × 100]

### 2.11. Guide RNA Target Intergenic Locus (ORF-157 and ORF-158)

The target sequence (5′-CGAGGAAAAGCTGTAGTTAT-3′) for guide RNA (gRNA) was analyzed by using online software (https://www.dna20.com/eCommerce/cas9/input accessed on 20 August 2019). The gRNA was constructed under the control of a U6 promoter in a lentiviral vector with antibiotic resistance to puromycin (Vector Builder, Shenandoah, TX, USA).

### 2.12. Donor Vector

The homologous region (HR) to the right (555 bp) and left (642 bp) of the intergenic locus between ORF-157 and ORF-158 (271 bp) were selected based on the ACAM2000™ DNA genome sequence (Genebank: AY313847). Multiple cloning sites were added at both ends of each HR to allow future insertion of any therapeutic gene. TurboFP635 was flanked by vaccinia virus early late promoter (pEL) and vaccinia termination signals. Three constructed fragments (HR-left, TurboFP635, and HR-right) were synthesized (GeneWiz, San Diego, CA, USA) and cloned into the pUC18 vector using an In-Fusion^®^ Cloning Kit (Takara Bio USA, Mountain View, CA, USA). The donor plasmid was confirmed by Sanger sequencing (Retrogen, San Diego, CA, USA).

### 2.13. Cas9HFc Vector

A plasmid containing the Cas9HF1 sequence, without nuclear localization, was synthesized by Vector Builder (Shenandoah, TX, USA) [29].

### 2.14. Transfection and Viral Infection

A total of 2 × 10^6^ CV-1 cells were seeded in a 6-well plate a day before transfection. Cells at 60–70% confluency were transfected with 1 µg each of plasmid encoding Cas9HFc and gRNA using 6 µL of TurboFectin 8.0 transfection reagent (Origene Technologies, Rockville, MD, USA) in 250 µL of opti-DMEM (Thermo Fisher Scientific, Waltham, MA, USA). At 24 h post-transfection, cells were infected with CAL1 VACV at an MOI of 0.02 in high glucose DMEM supplemented with 2% FBS. Two hours after viral infection, the cells were washed with PBS. 1.5 mL DMEM was added to the wells and the plate was incubated at 37 °C with CO_2_ for 30 min before being transfected with 2 µg of the donor plasmid generated above. The cells were further incubated at 37 °C with 5% CO_2_ and in a humidified atmosphere for 24 h. The mixture of infected/transfected cells was harvested and stored at −80 °C until the virus was screened and purified.

### 2.15. Virus Purification

The mixture of infected/transfected cells were thawed and then sonicated at maximum magnitude for 30 s, three times on/off on ice to release viral particle from the cells. 2 µL of released virus per plate was used to infect four confluent wells of CV-1 cells in 6-well plates. Two days after infection, 4–6 positive (red) plaques were picked up under 2x fluorescence microscopy and transferred into cryovials containing 200 μL serum-free DMEM. The purification process was repeated 2–4 times to obtain pure clones.

### 2.16. PCR Confirmation

To confirm the insertion of the transgene (TurboFP635) at the intergenic locus, a primer pair was designed to amplify the whole intergenic area with reverse (5′-GACGAAGAAGCAAGAGATTGTGT-3′) and forward (5′-ACCGTTTCCATTACCGCCA-3′) primers located on the left and right HR. The PCR products of the purified clones were sent out for Sanger sequencing (Retrogen, San Diego, CA, USA) for sequence confirmation.

### 2.17. Statistics

Statistical comparisons between 2 groups were performed using unpaired two-tailed Student’s t-tests with unequal variance. One-way or two-way ANOVA test was used for experiments with more than 2 groups. Statistical calculations were performed with GraphPad Prism 9.0.2 software (San Diego, CA, USA). *p*-values less than 0.05 were considered significant (* *p* < 0.05, ** *p* < 0.01, *** *p* < 0.001 and **** *p* < 0.0001).

## 3. Results

### 3.1. CAL1 Vaccinia Virus Derived from ACAM2000™ Is a Potent Oncolytic Virus and Preferentially Amplifies in Cancer Cells

As we previously described, ACAM2000™ vaccinia virus is a potent oncolytic virus with a confirmed safety profile in cancer patients. When ACAM2000™ was administrated in cancer patients, loaded into autologous Stromal Vascular Fraction (SVF) cells, we documented signals of efficacy, especially in combination with other immunotherapies [1]. In this study, we protect the smallpox vaccine ACAM2000 with an allogeneic AD-MSC to develop an off-the-shelf optimized stem cell-based oncolytic agent, called SNV1.

First, we amplified the selected vaccinia virus strain using CV-1 cells as a host cell line and named the resulting derivative CAL1. We used next generation sequencing (NGS) to determine if there were genetic differences after amplification of CAL1 from the parental virus. Our results indicated that when compared to the published ACAM1000/ACAM2000 sequence (Genbank AY313847), CAL1 (i) had a single nucleotide polymorphism (SNP) found within a non-coding region of the inverted terminal repeat (ITR) sequence at position 32 of the CAL1 genome, (ii) was shortened by 6 bps in the left ITR and (iii) was shortened by 197 bps in the right ITR. Those minor differences might be a consequence of using different sequencing technology. CAL1 vaccinia virus shared the same genome with ACAM2000 or ACAM1000 indicating high genetic stability using CV1 cells as a host cell line and as described before for ACAM2000 [13].

**Figure 1:**
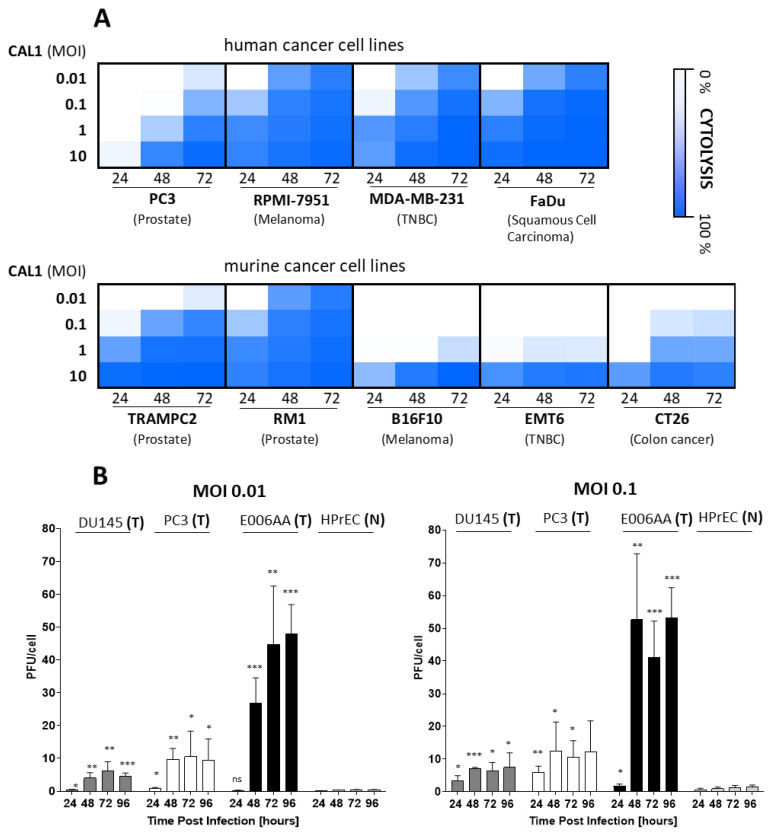
Cytolysis and tumor selective amplification of CAL1 vaccinia virus. (**A**) Cytolysis of human and mouse cancer cell lines post infection with CAL1 virus. Human cancer cell lines PC3 (Prostate cancer), RPMI-7951 (Melanoma), MDA-MB-231 (Triple negative breast cancer), FaDu (Head and Neck cancer/Squamous cell carcinoma) or mouse cancer cell lines TRAMPC2 (Prostate cancer), RM1 (Prostate cancer), B16F10 (Melanoma), EMT6 (Triple negative breast cancer), or CT26 (Colon Cancer) were seeded a day before and left untreated (control) or treated with CAL1 vaccinia virus at Multiplicity of Infection (MOI) of 0.01, 0.1, 1 or 10. CAL1-induced killing (cytolysis) was automatically analyzed using xCELLigence Real Time Cell Analysis Instrument (RTCA). The cytolytic activity at 24, 48 or 72 h was calculated as the percentage of cytolysis represented as heat map. Statistical significance was analyzed between all treated groups, 24, 48 ant 72 h, compared to control group for each individual cell line using two-way ANOVA test, * *p* < 0.05, ** *p* < 0.01, *** *p* < 0.001 (Table S3) (**B**) Tumor selective amplification of CAL1 Vaccinia virus. The human prostate cancer-derived cancer cell lines PC3, DU145, and E006AA, and the human primary prostate epithelial cell line HPrEC, were synchronously infected at a MOI of 0.01 or 0.1 with CAL1. Samples were collected at 24-, 48-, 72-, and 96-h post-infection and virus amplification was analyzed by plaque assay. T = human cancer cell line. N = normal human primary prostate epithelial cell line. Viral amplification at every time point for each tumor cell type was compared with HPrEC cells using unpaired 2-tailed Student’s *t* test (* *p* < 0.05, ** *p* < 0.01, *** *p* < 0.001) (n = 3), ns: no score.

Next, we examined the CAL1-induced cytotoxicity in multiple human and mouse cancer cell lines in vitro using real-time cell analysis (RTCA) system (xCelligence). Briefly, several concentrations of CAL1 virus were added to an xCelligence-well plate system containing human cancer cell lines: PC3 (Prostate cancer), RPMI-7951 (Melanoma), MDA-MB-231 (Triple negative breast cancer), FaDu (Head and Neck cancer/Squamous cell carcinoma), or murine cancer cell lines: TRAMPC2 (Prostate cancer), RM1 (Prostate cancer), B16F10 (Melanoma), EMT6 (Triple negative breast cancer), or CT26 (Colon Cancer). The resulting cytopathic kinetic curves were automatically recorded, and cytolysis was graphed at 24, 48 and 72 h post infection. CAL1 vaccinia virus induced dose- and time-dependent cell death in all human and murine cancer cell lines tested (Figure 1A). In addition, the difference in viral amplification between human prostate cancer cell lines PC3, DU145, and E006AA, and the human primary prostate epithelial cell line HPrEC, were analyzed. Cells were infected with CAL1 at Multiplicity of infection (MOI) of 0.01 and 0.1 and the viral amplification was examined at 24, 48, 72, and 96 h by plaque assay (Figure 1B). Our analyses indicated that all human cancer cell lines (PC3, DU145, and E006AA) supported higher virus amplification in comparison with the non-cancer cells (HPrEC). Specifically, the human cancer cell line E006AA showed the most prominent sensitivity to CAL1, with high viral amplification observed at the lowest MOI tested, 0.01. PC3 cells showed virus amplification levels similar to DU145 cells. In contrast, limited CAL1 amplification was observed in the HPrEC cells. These findings indicated significant preferential amplification of CAL1 in tumor derived cell lines as compared to primary cells derived from the same tissue of origin.

### 3.2. AD-MSCs Ad-Mscs Protect Oncolytic Viruses from Elimination by the Human Humoral Immunity

As described above, oncolytic virotherapy is a promising immuno-oncology approach that has not yet realized its full potential due to the rapid virus elimination by humoral immunity mediated by complement and neutralizing antibodies [30]. Most solid tumors express high levels of complement proteins in the tumor microenvironment [31], which leads to suppression of the therapeutic efficacy of oncolytic viruses even after i.t administration. Remarkably, complement-depleted mice contained up to 117-fold more live vaccinia viruses present in their tumors 48 h after i.t administration [30], indicating the need of protecting the virus even after i.t administration.

In our recent clinical trial, we showed that adipose-derived autologous stromal vascular fraction (SVF) cells have the potential to protect and deliver oncolytic vaccinia virus [1]. Here, we describe the development of an allogeneic cell-based platform, where the virus can be protected from humoral immunity inside the stem cells to minimize clearance by the immune system. This new platform was optimized to drastically enhance the therapeutic efficacy of intratumorally administered oncolytic viruses, creating an easy to manufacture off-the-shelf product. Specifically, AD-MSCs were loaded with CAL1 virus and cultured for 24 h. During the incubation period, vaccinia virus is amplified in the stem cells to reach ~10 plaque-forming units (PFU)/cell without altering cell viability. At thawing, SNV1 cells had a viability of 95.4% (Table S2). The AD-MSCs loaded with CAL1 were named SuperNova1 (SNV1) and were cryopreserved and stored at −80 °C. 

**Figure 2:**
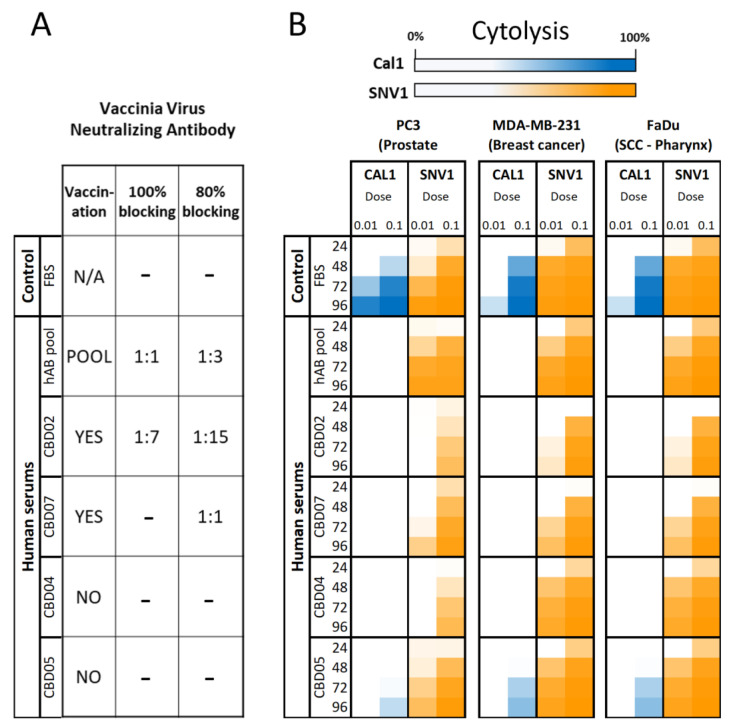
SNV1 Treatment Overcomes Human Serum Induced CAL1 Virus Inactivation. (**A**) Sera from different donors were explored for levels of neutralizing antibodies to CAL1 infectivity blockage before infection on monolayer CV1 cells. Percentage of oncolysis was real time measured using RTCA. Complete (100%) or partial (80%) blockage of the viral infectivity was analyzed. Number indicates serum dilution factors, (−) means no dilution, N/A = do not apply. POOL = Serotype AB pool. (**B**) Heatmap shows time courses in which cancer cells were killed by CAL1 and SNV1 at 24, 48, 72 and 96 h post infection in presence of 20% of human serum from AB pool or different donors with or without existing vaccinia virus neutralizing antibodies. CBD02, CBD07, CBD04, CBD05 are the individual human serum donors. Cytolysis was automatically analyzed by measuring cell impedance every 15 min using the RTCA MP Instrument. Results are the average of 4 replicate wells. RTCA = Real-time cell analysis; SNV1 = Supernova-1; SCC = squamous cell carcinoma.

Both CAL1 (naked virus) and SNV1 (virus-loaded stem cells) were tested for their ability to kill cancer cells in the presence of 20% human serum from donors with known vaccination statuses. Presence of neutralizing vaccinia antibodies in serum from different donors and Human AB pool was evaluated using real-time-cell-analysis as previously described [32] (Figure 2A). Thus, cancer cells PC3 (prostate cancer cells), MDA-MB-231 (breast cancer cells) and FaDu (head & neck cancer cells) were incubated with naked CAL1 virus or SNV1 in the presence of 20% human serum from vaccinated and unvaccinated healthy blood donors. PC3, MDA-MB-231 and FaDu cancer cells were seeded on electrode 96-well plates a day before and then treated with CAL1 or SNV1 at MOI of 0.01 and 0.1 in the presence of 20% of human serum from AB pool or different donors with or without existing vaccinia virus neutralizing antibodies (Figure 2B). We found that all serums from the vaccinated donors (AB pool, CBD02, CBD07) substantially inactivated naked CAL1 virus. Unvaccinated donor CBD05 and CBD04 also greatly inhibited the therapeutic potential of CAL1 (Figure 2B). Importantly, heat inactivation of serum from donors CBD05 or CBD04 eliminated serum-induced vaccinia virus inactivation, emphasizing the critical role of human complement-induced inactivation (Table S1). In contrast to the naked CAL1, SNV1 demonstrated potent resistance to human serum inactivation of CAL1 in all tested donors, regardless of their vaccination status. 

MOI for SNV1 is calculated by considering one stem cell loaded with CAL1 viruses (SNV1) as 1 infection unit, as cells are added as infection units and not free viruses to treat cells in culture. The SNV1 lot was released by quantifying the viral load per cell by plaque assay as described in the materials and methods section. Each SNV1 cell contained approximately 10 PFU and viability was higher than 90%. In other words, if we seed 10^4^ cancer cells per well, SNV1 treatment at MOI 0.1 (MOI based on infection units) is equivalent to 10^3^ SNV1. Similarly, SNV1 treatment at MOI 0.01 is equivalent to 10^2^ SNV1. In this specific experiment one (1) SNV1 contains about ten (10) PFU CAL1 virus, thus the absolute CAL1 virus input in the setting of naked CAL1 (MOI 0.1) was equivalent to SNV1 (MOI 0.01). The results in Figure 2 showed significant benefits of using SNV1 to target three human cancer cell lines at MOI 0.01 (MOI based on infection units), compared to naked CAL1 virus at MOI 0.1 in the presence of different human serums. It is important to note that SNV1 do not only protect the viruses against humoral immunity but, the oncolytic vaccinia virus amplifies inside the stem cells allowing a more potent therapy as shown in SNV1 MOI 0.1 condition. SNV1 is not only a delivery vehicle but a more potent therapy.

### 3.3. The Use of AD-MSC Significantly Increases the Therapeutic Efficacy of CAL1 in Tumor-Bearing Mouse Models

**Figure 3:**
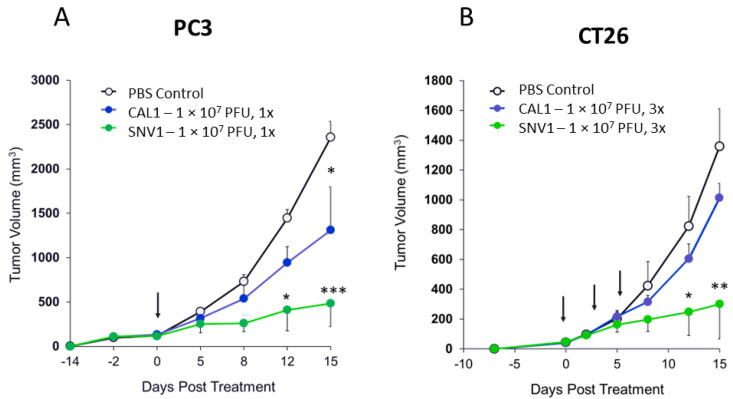
Inhibition of tumor growth after treatment with SNV1 or with CAL1 naked vaccinia virus. (**A**) Athymic nude male mice aged 4–6 weeks were inoculated s.c. with aggressive metastatic prostate cells (PC3) in the right flank. When tumors reached about 200 mm^3^ animals were i.t treated (day 0, arrow) with a single dose of 1 × 10^7^ PFU of CAL1 vaccinia virus or 1 × 10^6^ SNV1 cells (10 PFU/cells) (n = 10). (**B**) Female BALB/c mice were inoculated s.c. with 2.5 × 10^5^ murine colon cancer cells (CT26). When tumor sizes were around 50 mm^3^, mice were i.t treated with 1 × 10^7^ PFU of CAL1 vaccinia virus or 1 × 10^6^ SNV1 cells three times (arrows), every 3 days (n = 6–7) (day 0, marks as the first treatment). Data is presented as geometric mean with SEM. PBS treated group served as a control. Tumor size was measured two times per week. Statistical significance was analyzed between treated groups compared to control group using one-way ANOVA test, * *p* < 0.05, ** *p* < 0.01, *** *p* < 0.001.

We further analyzed the therapeutic potential of SNV1 in comparison to naked CAL1 vaccinia virus in the human prostate tumor-bearing immunocompromised mouse model PC3, and in the murine colon tumor-bearing immunocompetent mouse model CT26. Two different models were chosen based on different capacity of the CAL1 virus to kill the tumors (higher in human PC3 and lower in mouse CT26 cancer cell lines as shown in Figure 1) and based on the hypothetical capacity of immune system to facilitate clearance of SNV1 treatment (low in immunocompromised athymic nude mice and higher in immunocompetent BALB/c mice) Frozen stocks of SNV1 were generated and stored at −80 °C or in liquid nitrogen. Tumors were generated by subcutaneous inoculation of PC3 (human prostate cancer cell line) or CT26 (mouse colon cancer cell line) cells into the right flank of athymic nude or BALB/c mice, respectively. When tumor sizes reached approximately 200 mm^3^ (PC3) or 50 mm^3^ (CT26), respectively. The mice were treated once (PC3) or three times (CT26) via intratumoral injections of 1 × 10^7^ PFU CAL1 naked virus or with 1 × 10^6^ SNV1 cells containing CAL1 virus (equivalent to 1 × 10^7^ PFU). We found that SNV1 drastically inhibited tumor growth in comparison to either the naked virus or PBS controls of both PC3 (Figure 3A) and CT26 (Figure 3B) tumor models. Interestingly, although both treatments had equivalent number of viral particles, the SNV1 treatment was significantly more efficacious in delaying tumor growth rates, confirming the higher therapeutic potential of SNV1 when compared with naked virus. Three administrations were used in immunocompetent mouse models mimicking a human clinical scenario of repeated doses.

### 3.4. CAL1 Vaccinia Virus as a Backbone to Express Therapeutic Genes in Cancer Cells

**Figure 4:**
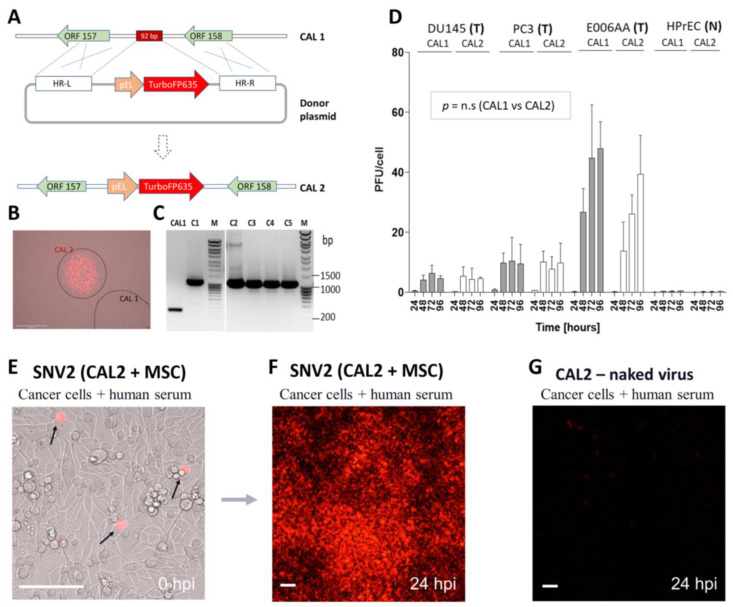
CAL1 vaccinia virus as a backbone to express therapeutic genes in the context of MSC delivery to cancer cells. (**A**) Strategy to generate new armed virus using CAL1. The intergenic locus in CAL1 genome was cut by Cas9HFc/gRNA, which trigger homologous recombination between CAL1 and the donor vector. As an example, TurboFP635 expression cassette from the donor vector was exchanged for the 92 bp fragment in the intergenic locus of CAL1, resulting in the recombinant virus, CAL2. (**B**) Generation of CAL2 virus. During clonal selections of CAL2, the recombinant CAL2 virus expressing TurboFP635 (red) was isolated from parental CAL1 virus (white). (**C**) Verification of generation of armed-vaccinia virus by PCR. Genomic DNA from six selected clones was used as template for PCR amplification using primers specific to the intergenic region where the TurboFP635 expression cassette was inserted. The uncropped DNA Figure can be found as Figure S1 in Supplementary Materials. The recombinant clones (C1–C6) show a PCR band with a molecular weight higher than 1000 bp while the parental CAL1 virus was only 230 bp. (**D**) Comparison of CAL1 and CAL2 tumor preferential viral amplification. The prostate cancer derived human cancer cell lines PC3, DU145, and E006AA, and the human primary prostate epithelial cell line HPrEC were synchronously infected at a MOI of 0.01 with CAL1 or CAL2. Samples were collected at 24-, 48-, 72-, and 96-h post-infection and virus amplification was analyzed by plaque assay. T = human cancer cell line. N = normal human primary prostate epithelial cell line. CAL1 Viral amplification at every time point and for every cell line was compared with CAL2 viral amplification. *p* = n.s (no significant) unpaired 2-tailed Student’s *t* test (n = 3). (**E**–**G**) Therapeutic potential of SNV2. PC3 prostate cancer cells were infected with SNV2 in presence of 20% human serum. (**E**) Gene of interest (for example TurboFP365) was present at time of treatment in SNV2 cells (black arrows). Picture shows overlay of bright field showing monolayer of PC3 cells and SNV2 expressing TurboFp635 fluorescent protein detected using Invitrogen EVOS FL Auto 2 Cell Imaging System. (**F**) Twenty-four (24) hours post treatment with SNV2, massive expression of CAL2-encoded TurboFp635 was found in cancer cells despite presence of 20% human serum, whereas very limited TurboFp635 expression was noted in CAL2 naked virus-treated cells due to serum-induced inactivation of CAL2 virus (**G**).

Vaccinia virus has been used as a platform to express therapeutic genes in cancer cells. The therapeutic genes (i.e., cytokines, chemokines, therapeutic antibodies, or enzymes) are generally inserted into the viral genome in a manner that disrupts the expression of one or more viral genes, thereby attenuating the virus and improving tumor selectivity [33,34]. However, such attenuated viruses might lose the capacity to efficiently replicate in vivo. In naturally attenuated viruses, further attenuation can decrease their therapeutic potential. We have found a new site in the CAL1 genome where therapeutic genes can be inserted without altering functional viral ORFs in the resulting recombinant virus. As a proof of concept, we sought to engineer a virus suitable for the visualization of virus-infected cells in vivo. Briefly, a 92 bp middle fragment that was found in the intergenic area between ORF-157 and ORF-158 (position 138,314 to position 138,407 Genebank AY313847) was replaced by TurboFP635 (Figure 4A) using the CRISPR/Cas9HF system with high fidelity cytosolic Cas9 protein [29]. Microscopy analysis of clones from CV-1 infected cells showed that TurboFP635 was successfully inserted into the engineered CAL2 recombinant vaccinia virus (Figure 4B). PCR (Figure 4C) and Sanger sequencing confirmed the insertion of the TurboFP635 gene into the resulting recombinant virus, which was named CAL2. Subsequently, virus amplification was compared between CAL1 and CAL2 infected cells (Figure 4D). The cell lines used included the prostate cancer derived human cancer cell lines PC3, DU145, E006AA, and the human primary prostate epithelial cell line HPrEC. We found that the human cancer cell lines showed very high virus amplification with levels of PFU per cell of >10 at 72 h after infection. No significant differences in virus amplification were noted in any of the three human cancer cell lines. HPrEC line showed minimal virus amplification of CAL1 and CAL2 as compared to the human cancer cell lines, similarly to our previous findings (Figure 1B). Overall, we have demonstrated that the introduction of potentially therapeutic genes at the intergenic site between ORF-157 and ORF-158 does not compromise the natural tumor selectivity of the virus or its amplification potential in cancer cells.

Next, we compared the capacity of unprotected CAL2 virus or SNV2 (CAL2 loaded into AD-MSC) to infect and expressed TurboFP635 (viral-encoded payload) in PC3 prostate cancer cells in the presence of 20% human AB serum. Results showed the expression of was immediately detected in the carrier stem cells at the time of treatment with SNV2 (Figure 4E). Twenty-four hours post treatment with SNV2, high expression of TurboFp635 was found in PC3 cancer cells despite the presence of 20% human serum, indicating that CAL2 virus has been protected by AD-MSC (Figure 4F). On the other hand, very limited expression of TurboFP635 was found after treatment with CAL2 naked virus due to serum-induced inactivation of the virus (Figure 4G). The protection of SNV to human serum clearance was also demonstrated in different tumor cell lines (Video S1–S2). Taken together, our results indicate that CAL1 can be used as a backbone to engineer recombinant viruses armed with therapeutic genes that could be delivered using SNV to be therapeutically effective in patient

## 4. Discussion

Several unmodified naturally tumor-selective vaccinia viruses have been used in the past as anti-cancer therapies. Dryvax^®^, a heterogeneous pool of VACV clones used as a smallpox vaccine, has also demonstrated clinical safety, feasibility, and therapeutic efficacy against cancer [35,36,37,38,39]. However, VACV clones with varying degrees of virulence were present in Dryvax^®^ increasing the risk, especially when using this vaccine in immuno-compromised cancer patients [14,40,41].

In the early 2000s, growing concerns over bioterrorism brought smallpox vaccination back to the forefront. In 2008, the FDA approved ACAM2000™ a plaque-purified derivative of Dryvax^®^ manufactured in vaccine-certified cell culture as smallpox vaccine [15,40]. Here, we showed that ACAM2000 vaccinia virus could be repurposed as an anti-cancer treatment if protected, delivered, and potentiated with allogeneic AD-MSCs.

In the past, efforts have been made to reduce the therapeutic risks associated with the use of VACV with an unknown safety profile via genetic modifications eliminating certain elements of the viral sequence. An alternative strategy to generate a safer VACV for use in cancer therapy is to select a naturally occurring clone with a significantly reduced virulence, improved safety, and without the need of further attenuation. Thus, we selected ACAM2000™ vaccinia virus, a potent TK-positive oncolytic virus strain with a confirmed safety profile in non-cancer [15] and cancer patients [1] to develop an off-the-shelf allogenic stem cell-based platform for delivery of oncolytic virus therapy. To develop this new therapeutic, we first amplified and generated a derivative vaccinia virus named CAL1 using CV1 cells as a host cell line known to offer high yields during manufacturing [42,43] CAL1 derived from CV-1 cells had same genomic sequence as ACAM1000 and ACAM2000 confirming the described genomic stability of this VACV strain [13].

Importantly, CAL1 was able to selectively infect, replicate, amplify and lyse multiple mouse and human cancer cell lines. However, as shown for other oncolytic viruses, CAL1 can be quickly inactivated by innate and humoral immune system. The complement system and/or neutralizing antibodies act by inactivating both intravenously and intratumorally administered viruses, creating an immune barrier to virotherapy [30]. Oncolytic virotherapy is a promising immuno-oncology approach that has not yet realized its full potential due to the rapid virus elimination by the complement system and neutralizing antibodies [30]. Most solid tumors express high levels of complement proteins in the tumor microenvironment (TME) [31], which leads to strong inhibition of oncolytic viruses and minimal therapeutic efficacy even after i.t administration. Remarkably, complement-depleted animals contained up to 117 fold more live vaccinia viruses present in their tumors 48 h after i.t administration [30].

Although there are known limitations in the use of animal models to explore the therapeutic potential of human cancer immunotherapeutic in the context of human clinical barriers [30,44,45] our ex vivo data shows oncolytic viruses can be protected from the patients’ immune system by loading the virus into stem cells. Stem cells are known for their ability to secrete anti-inflammatory cytokines, to home the injured or inflamed tissues and to potentiate oncolytic viruses [22,46]. Stem cells originating from several tissue sources have been used to deliver oncolytic viruses, including: menstrual blood [47,48,49], neural tissue [21,50,51,52], bone marrow and umbilical cord blood [53,54,55]. Autologous stem cells have been used to optimize systemic delivery of oncolytic viruses. Here, we describe the development of an allogeneic stem cell-based platform, where the virus can be protected and potentiated, leading to an enhanced therapeutic efficacy following i.t administration.

Direct i.t administration of immune oncology agents often offers a better and more successful translation to the clinic since it efficiently targets the tumor and avoids the significant dilution associated with intravenous approaches [25] Earlier treatment using i.t administration of oncolytic virus was shown to activate tumor infiltrating T cells leading to a reduced tumor recurrence [56]. Intratumoral immunotherapies using oncolytic viruses such as herpes virus T-VEC, herpes HF10, or vaccinia virus JX-594 are actively adopted in clinical trials for treatment of solid tumors. Our data indicate that oncolytic virus therapies lead to a better therapeutic response after i.t administration when delivered via stem cells. This approach has the further potential to improve the therapeutic efficacy of existing oncolytic viruses already being tested in numerous clinical trials. Of note, a recent clinical study, using allogeneic neural stem cells delivering the oncolytic adenovirus CRAD-s-PK7 has shown acceptable safety profile in patients with newly diagnosed high-grade glioma [52].

To increase the therapeutic potential and precision of oncolytic VACV, researchers have engineered viruses to deliver various therapeutic genes, but so far, the efforts have resulted in highly attenuated recombinant viruses due to the disruption of essential viral genes by the inserted transgenes [19,30]. Our findings show that TurboFP635, a far-red fluorescent protein, can be successfully engineered into CAL1 without genetically disrupting known viral ORFs. Specifically, an insertion of the TurboFP635 gene was made at a 92 bp middle fragment between ORF-157 and ORF-158. Many attempts to engineer different types of oncolytic viruses to express various therapeutic genes have been made by multiple research groups. However, the expression of those therapeutic proteins might be limited in clinical settings due to the efficient viral clearance by the patient’s immune system. Interestingly, when recombinant virus was delivered using the allogeneic stem cell platform described in this study, the stem cells not only carried the virus but also expressed the therapeutic proteins encoded by the virus, which has the potential to express reporter proteins and/or therapeutic agents. 

## 5. Conclusions

We demonstrated that CAL1 possesses tumor selectivity, induces tumor regression, and can serve as a backbone for the introduction of therapeutic genes. Moreover, CAL1 can be loaded into AD-MSCs to generate SNV1. The SNV1 stem cell-based platform protects and potentiates oncolytic vaccinia virus by circumventing humoral innate and adaptive immune barriers, resulting in enhanced anti-tumor effects in vivo. Importantly, SNV1 can produce instantly active viral particles for faster and more effective targeting of cancer cells and simultaneous release of therapeutic proteins encoded by the virus, which can immediately modify the tumor microenvironment. This platform is designed to work as a stand-alone treatment or in combination with systemic immunotherapies that require reconfiguration of the tumor microenvironment, such as checkpoint inhibitors or cell therapies aimed to target solid tumors.

## 6. Patents

The authors declare that a patent application entitled “enhanced systems for cell-mediated oncolytic viral therapy” has been filed on 6 November 2019 by the inventors Antonio Fernandez Santidrian, Duong Hoang Nguyen and Dobrin Draganov (WO2020097269A1).

## Data Availability

The data presented in this study are available in this article and Supplementary Materials.

## Supplementary Materials

The following supporting information can be downloaded at: https://www.mdpi.com/article/10.3390/cancers14246136/s1, Figure S1: Verification of generation of armed-vaccinia virus by PCR (uncropped agarose gel), Table S1: Characterization of serums from different donors; Table S2: SNV1 viability and cell count; Table S3: Two-way ANOVA test; Video S1: SNV protects oncolytic viruses from elimination by the human humoral immunity in killing MDA-MB-231 cancer cells; Video S2. SNV protects oncolytic viruses from elimination by the human humoral immunity in killing FADU cancer cells.
