# Supplementary material for: Development of Allogeneic Stem Cell-Based Platform for Delivery and Potentiation of Oncolytic Virotherapy
Source: Cancers (Basel). 2022 Dec 13;14(24):6136. doi: 10.3390/cancers14246136 (PMC9777144; doi:10.3390/cancers14246136)
Supplement: Supplementary file 1 [file cancers-14-06136-s001.zip › cancers-1967088-supplementary/cancers-1967088-supplementary.pdf]

Supplementary Materials: Development of Allogeneic Stem Cell-Based Platform for Delivery and Potentiation of Oncolytic Virotherapy

Duong Hoang Nguyen, Thomas Hermann, Barbara Härtl, Dobrin Draganov, Ivelina Minev, Forrest Neuharth, Alberto Gomez, Ashley Alamillo, Laura Edith Schneider, Daniela Kleinholz, Boris Minev and Antonio F. Santidrian

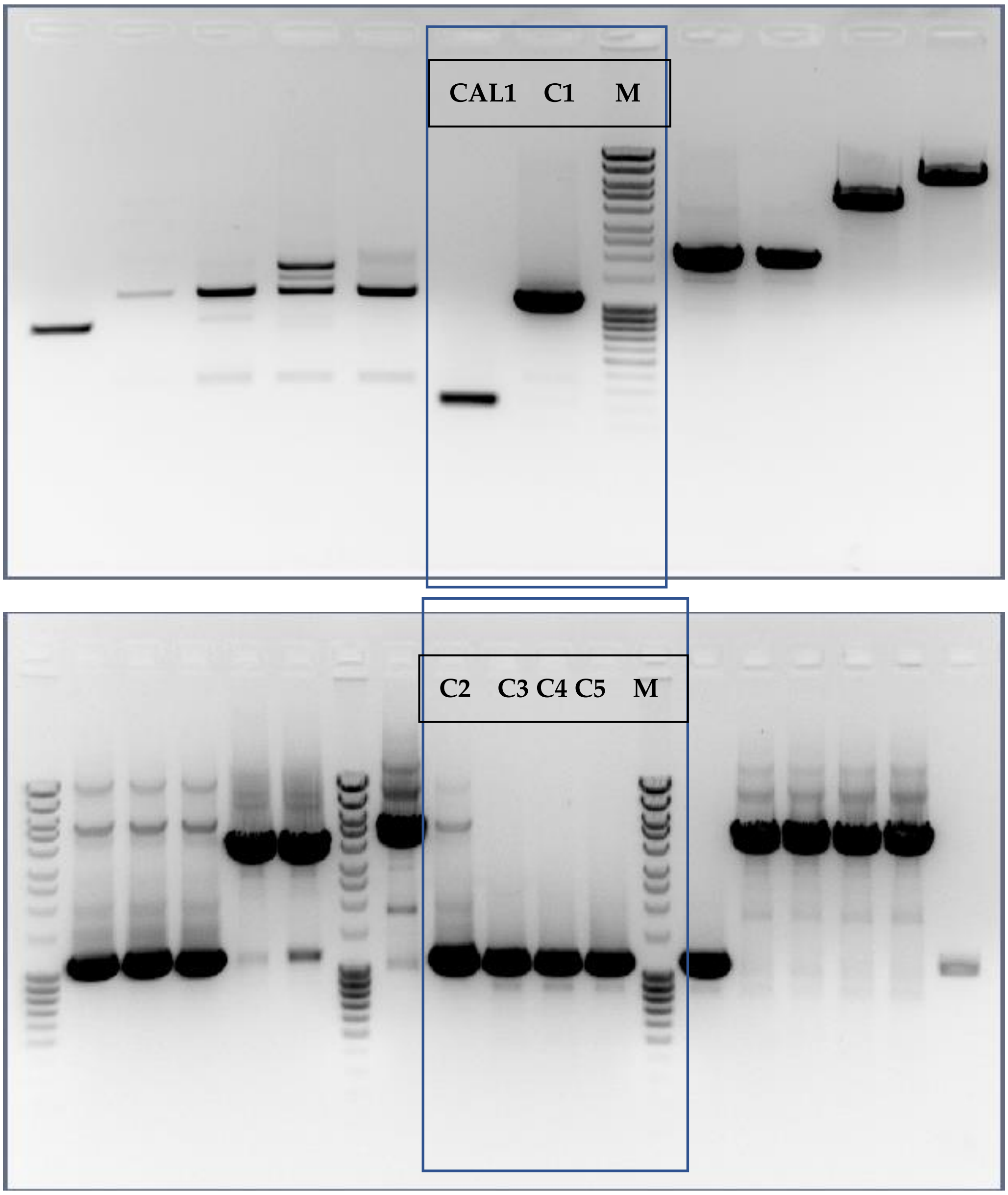

Figure S1. Verification of generation of armed-vaccinia virus by PCR (uncropped agarose gel). Genomic DNA from six selected clones was used as template for PCR amplification using primers specific to the intergenic region where the TurboFP635 expression cassette was inserted. The recombinant clones (C1–C6) show a PCR band with a molecular weight higher than 1000 bp while the parental CAL1 virus was only 230 bp. Note: other lanes (outside of the boxes) are unrelated with the study presented in the manuscript.

Table S1. Characterization of serums from different donors.

Donor	Blood Type	Smallpox Vaccination	% CAL1 recovery in 90% serum	% CAL1 recovery in 90% heat Inactivated serum
CBD-04	AB	No	1.20%	93.20%
CBD-05	B+	No	2.00%	77.30%
CBD-07	A+	Yes	0.50%	40.90%
CBD-02	B+	Yes	0.30%	18.00%
hAB pool	AB	N/A	29.20%	48.70%

N/A; do not apply, unknown.

Serums or heat-inactivated serum from healthy donors used in the study, with known vaccination status against smallpox, were characterized based on capacity to inhibit vaccinia virus CAL1. Human serum donors were heat-inactivated at 65°C for 30 minutes. In brief, 5×10^4 PFU CAL1 viruses were incubated with 100 uL containing 90% serums or heat-inactivated serum from healthy human donors CBD02, CBD07, CBD04, CBD05, pooled AB serum (hAB pool) or serum free media for 1 hour at 37°C with agitation every 15 minutes. Once the incubation was completed, the presence of infectious (active) vaccinia virus was analyzed by plaque assay as described in Materials and Methods. Supplementary Table 1 shows % of CAL1 vaccinia virus recovered in all conditions compared to serum free media condition.

Data shows that all non-heat inactivated serums from all single donors drastically inhibited vaccinia virus CAL1. Importantly, heat inactivation of serums from non-vaccinated donors CBD05 or CBD04 did not inactivated vaccinia virus when compared with non-heat inactivation, emphasizing the critical role of human complement-induced vaccinia virus inactivation. Furthermore, heat inactivation of serum from vaccinated donors CBD07 or CBD02 were still able to inactivate vaccinia virus due to the presence of neutralizing antibodies.

Table S2. SNV1 viability and cell count.

NC200	SNV1 count after thawing		
	**Total (cells/ml)**	**Viability (%)**	**Diameter**
Count 1	6.82×10^6	95.00%	13.30
Count 2	6.90×10^6	96.30%	13.30
Count 3	6.78×10^6	94.90%	14.50
Average	6.83×10^6	95.40%	13.70

The viability and cell count of SNV1 was determined using automate NucleoCounter® NC-200™. Briefly, a frozen SNV1 was thawed in water bath for 1–2 minutes and diluted in CSB buffer (https://www.stemcell.com/products/cryostor-csb.html) to have concentration around 1-2e6 cells per ml. Three aliquots of 200 µl of SNV1 were used for NC200 count using Viability and cell count – Aggregated program (following manual protocol) https://chemometec.com/products/nucleocounter-nc-200/

Supplementary Tables S3 are provided separately, attached as an Excel file.

Video S1. SNV protects oncolytic viruses from elimination by the human humoral immunity in killing MDA-MB-231 cancer cells. Human triple negative breast cancer MDA-MB-231 (10x magnification) cells were seeded in 96 well plates for 24h. when cells were about 80-90% confluent, cells were infected with CAL2 virus (expressing red fluorescent protein) or SNV2 (stem cells loaded with CAL2) at MOI of 0.1 in the presence of 20% human serum positive with vaccinia virus antibody. SNV2 cells were stained with CellTracker™ Green CMFDA Dye (Thermofisher) before adding to the cancer cells. The cells were captured real-time for bright field, red (CAL2 virus) and green (SNV2) fluorescent channels using Cytation 7 imaging system (Agilent).

Video S2. SNV protects oncolytic viruses from elimination by the human humoral immunity in killing FADU cancer cells. squamous cell carcinoma FADU (4x magnification) cells were seeded in 96 well plates for 24h. when cells were about 80-90% confluent, cells were infected with CAL2 virus (expressing red fluorescent protein) or SNV2 (stem cells loaded with CAL2) at MOI of 0.1 in the presence of 20% human serum positive with vaccinia virus antibody. SNV2 cells were stained with CellTracker™ Green CMFDA Dye (Thermofisher) before adding to the cancer cells. The cells were captured real-time for bright field, red (CAL2 virus) and green (SNV2) fluorescent channels using Cytation 7 imaging system (Agilent).

Video S1-S2 are provided separately, attached as .mp4 files.
